# Bayesian methods for expression-based integration of various types of genomics data

**DOI:** 10.1186/1687-4153-2013-13

**Published:** 2013-09-21

**Authors:** Elizabeth M Jennings, Jeffrey S Morris, Raymond J Carroll, Ganiraju C Manyam, Veerabhadran Baladandayuthapani

**Affiliations:** 1 Department of Statistics, Texas A&M University, College Station, TX 77843, USA; 2 Department of Biostatistics, UT M.D. Anderson Cancer Center, Houston, TX 77030, USA; 3 Department of Bioinformatics and Computational Biology, UT M.D. Anderson Cancer Center, Houston, TX 77030, USA

**Keywords:** Bayesian modeling, Genomics, Hierarchical models, Integrative analysis, Shrinkage priors

## Abstract

We propose methods to integrate data across several genomic platforms using a hierarchical Bayesian analysis framework that incorporates the biological relationships among the platforms to identify genes whose expression is related to clinical outcomes in cancer. This integrated approach combines information across all platforms, leading to increased statistical power in finding these predictive genes, and further provides mechanistic information about the manner in which the gene affects the outcome. We demonstrate the advantages of the shrinkage estimation used by this approach through a simulation, and finally, we apply our method to a Glioblastoma Multiforme dataset and identify several genes potentially associated with the patients’ survival. We find 12 positive prognostic markers associated with nine genes and 13 negative prognostic markers associated with nine genes.

## 1 Introduction

**Figure 1:**
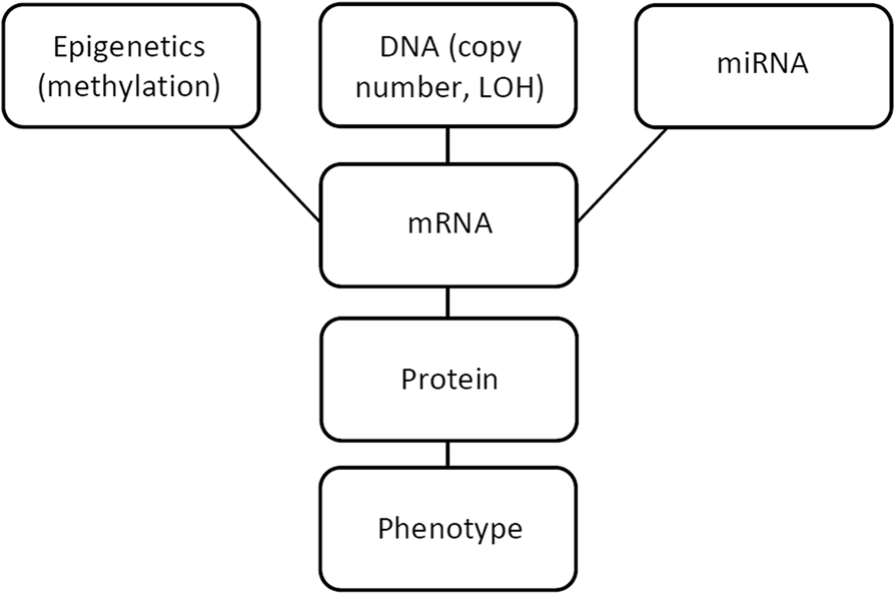
**Platform relationships.** Schematic representation of the multiple molecular platforms and their biological relationships.

The central dogma of molecular biology summarizes the steps involved in the passage of genetic information at a molecular level: DNA is transcribed to messenger RNA (mRNA), which is then translated to a protein, which carries out a specific action in an organism. In addition, there are also other alterations and interferences, such as epigenetic factors, that can occur at the DNA and/or mRNA levels which affect the ultimate expression of a given gene. In this paper, we consider methylation (which occurs at the DNA level and typically results in a silencing of the gene), copy number (which describes an attribute at the DNA level that affects mRNA expression), and mRNA expression (which affects protein expression); these subsequently affect a clinical phenotype (e.g., survival) (see Figure 1). In addition, it is believed that the mechanism of cancer development is complex and involves multiple genes [1]. It is known that genes interact and are related through certain pathways, and in this paper, we focus on genes from important signaling pathways that influence cancer progression and development [2].

Current technologies allow us to obtain data from the above-mentioned platforms (and many others) for each gene involved in the investigations. The Cancer Genome Atlas (TCGA) is a project that began in 2006 to gather comprehensive genomic data using multiple platforms on over 20 types of cancer [3]. The increasing availability of such data has motivated the development of methods that seek to improve estimation and prediction regarding genomic effects on cancer outcomes by integrating data from multiple platforms in a single analysis. The incorporation of information from more than one platform has the potential to increase power and lower false discovery rates in identifying markers related to clinical outcomes for cancer patients [4]; such improvements would deepen our understanding of how cancer develops and spreads, offering researchers valuable insight regarding the development of drugs and procedures intended to prevent or inhibit cancer development.

Some integration techniques consider different platforms sequentially and then draw conclusions from the combination of results. For example, the TCGA Research Network performed a large-scale study of ovarian cancer data, including specific platforms such as gene mutations, copy number, mRNA expression, miRNA expression, and DNA methylation. Within each platform, they compared normal and tumor cells to identify significant genes and combined the information obtained from different platforms to understand the deeper biology behind the cancer mechanisms, including gene interactions. Using the prevalence of significant genes, they also identified influential pathways, including the RB1 and PI3K/RAS pathways [5]. TCGA Research Network conducted a similar style study on Glioblastoma Multiforme (GBM) data and, among other things, discovered a previously unknown link between MGMT methylation and the mutation spectra of mismatch repair genes through the integration of mutation, methylation, and clinical treatment data [6]. These methods provide insight into the roles and interactions of genes as related to the development and outcome of the disease.

Another type of integrative method proposes incorporating multiple platforms in a single model. Such approaches must face the challenges of high dimensionality and complex biological relationships both within and between platforms. One such approach is iCluster, proposed by Shen et al., which is a joint latent variable model-based clustering method that integrates data from multiple genomic platforms to cluster samples into subtypes. iCluster achieves reduced dimension of the data, and it is shown to identify potentially novel subtypes of breast cancer and lung cancer [7]. However, this method does not directly model the biological relationships among platforms; in addition, it is an unsupervised method, while our approach is supervised. Tyekucheva et al. suggest a method that includes multiple platforms as predictors in a logistic regression model (with phenotype as the response), and they show that incorporating multiple platforms yields more power to detect differentially expressed genes than approaches that only use a single platform [8]. As with iCluster, this approach accounts for dependence between platforms, but it does not directly take into account their biological relationships.

Another method, proposed by Lanckriet et al., first represents data from each platform (such as primary protein sequence, protein-protein interaction, and mRNA expression) via a kernel function and then combines the kernels in a classification model (predicting, for example, protein type). It is shown that this method outperforms methods based on a single kernel from any one data platform [9]. However, this method does not directly model the relationships among the platforms, and kernel representations of the marker effects on the clinical outcomes are not directly interpretable. Liu et al. suggest another approach that integrates clinical covariates and multiple gene expressions (from a common pathway) to predict a continuous outcome through a semiparametric model; the covariates are modeled parametrically, and the pathway effect is modeled through least squares kernel machines (LSKM) (either parametrically or not). The covariate as well as pathway effects can be estimated, and the pathway effect can be tested for significance. The nonparametric LSKM regression allows for complicated interactions between genes [10], but this method only incorporates a single genomic platform (and accounts for its internal biological relationships). Recently, Wang et al. proposed an integrative Bayesian analysis of genomics data (iBAG) framework that models the biological relationships between two platforms [4]. This approach involves a global gene search and uses variable selection via the Bayesian lasso-based shrinkage priors to deal with the high dimensionality of the data.

In this paper, we introduce a generalized version of iBAG that integrates data from an arbitrary (multiple) number of genomic platforms using a hierarchical model that incorporates the biological relationships among them. We focus our analysis on genes from several important cancer signaling pathways and integrate mRNA, methylation, and copy number data to predict survival in GBM patients. In addition, we reduce dimension by regressing the clinical outcome on latent scores of the platforms (see Section 2.1 for details). To improve effect size estimation and to achieve sparsity, we use a Normal-Gamma (NG) prior for the effects, which increases flexibility in the estimation as compared to the Laplace prior of the Bayesian lasso [11] (see Section 2.2 for further discussion). Section 3 illustrates our methodology on a synthetic example; analysis of GBM data is presented in Section 4; and conclusions are drawn in Section 5.

## 2 A multivariate iBAG model

Our construction of a multivariate iBAG model employs a two-component hierarchical model where the first component can be considered as the *mechanistic model* and the second can be considered as the *clinical model*. In the first stage mechanistic model, we partition each gene’s expression into the factors explained by methylation, copy number, and other (unknown/unmeasured) causes using a principal component-based regression model. Subsequently, we include these factors as predictors in the second stage clinical model, thus finding not only those genes whose expression is directly related to clinical outcome, but also expression effects driven by methylation, copy number, or other mechanisms. We explain the construction of each of these components below.

### 2.1 Mechanistic model

Let *n* = number of patients, *J* = number of platforms being integrated, and *p*_*j*_ = number of genes from platform *j*. The mechanistic model for each gene can be expressed as:

$${\text{mRNA}}_{i}={\text{M}}_{i}+{\text{CN}}_{i}+{\text{O}}_{i},$$

where each of the terms are defined as follows: 

• mRNA_*i*_ is the level of gene expression for gene *i* (where *i* = 1,…,max(*p*_*j*_); *j* = 1,…,*J*) and is of dimension (*n* × 1).

• M_*i*_ is the part of gene_*i*_ expression that is attributed to methylation, and is of dimension (*n* × 1). Specifically, M_*i*_ is the product of some methylation predictor and a fitted coefficient. Details are below.

• CN_*i*_ is the part of gene_*i*_ expression that is attributed to changes in copy number, and is of dimension (*n* × 1). Specific calculation is similar to M_*i*_ – see below.

• O_*i*_ represents the ‘other’ (remaining) part of the gene expression that is explained by something other than methylation or copy number, and is of dimension (*n* × 1).

Since the raw methylation and copy number data for any given gene can contain multiple (up to 40 in our data) values from different markers within that gene, to estimate each of the components M_*i*_, CN_*i*_, and O_*i*_, we first carry out two principal component analyses (PCA) for gene_*i*_: one each for the methylation and copy number data, and in each case, we keep the number of principal components that retain ≥ 90*%* of the total variation. We then regress mRNA_*i*_ on the methylation and copy number PC scores. We use the estimated pieces and the corresponding residuals from this regression to estimate the vectors $M_{i}=\sum_{k=1}^{K}X_{i,k}^{M}B_{k}^{M}$ (where $X_{i,k}^{M}$ is the methylation value for gene *i* with *K* = 1 if there is only one methylation marker for that gene, or the methylation score for principal component *k* for gene *i* if there are multiple methylation markers for gene *i*, and $B_{k}^{M}$ is the vector of regression coefficients), ${\text{CN}}_{i}=\sum_{r=1}^{R}X_{i,r}^{\text{CN}}B_{r}^{\text{CN}}$ (where $X_{i,r}^{\text{CN}}$ is the copy number value for gene *i* with *R* = 1 if there is only one copy number marker for that gene, or the copy number score for principal component *r* for gene *i* if there are multiple copy number markers for gene *i*, and $B_{r}^{\text{CN}}$ is the vector of regression coefficients), and O_*i*_ = residuals. This process is repeated for each gene independently.

### 2.2 Clinical model

The clinical model component of our construction relates the effect of the mechanistic parts of the genes (as estimated above) to a clinical outcome of interest (e.g., survival, in our context) and can be written as:

$$Y=M\beta_{1}+\text{CN}\beta_{2}+\text{O}\beta_{3}+\varepsilon ,$$

where *Y* denotes the clinical outcome, ***β***_*j*_ are the effects of platform *j* on *Y*, and ***ε*** is the error term. The covariates in the model {M, CN, O} are the vectorized gene expression effects attributed to methylation, copy number, and other sources, respectively, and are estimated from the mechanistic model. In essence, our clinical component jointly (additively) models the effects of all the gene expressions and their components - derived from different sources (methylation/copy number) - in a unified manner. When the clinical response is survival, we use an accelerated failure time (AFT) model, taking *Y* to be log(survival) [12].

Our goal is to find a list of significant genes that affect the outcome via the various mechanisms; hence, efficient estimation of ***β*** = {***β***_1_,***β***_2_,***β***_3_} is of primary interest. One route would be to simply fit a least squares regression to estimate the parameters. However, the number of predictors is large compared to the number of samples, and, more importantly, we expect our solution to be very sparse since only a few genes will be related to clinical response; hence, least squares would overfit the data and yield less accurate results as compared to approaches that induce sparsity by shrinkage/penalization. We illustrate this fact in our simulation in Section 3.

To induce shrinkage/penalization, we follow a Bayesian approach and specify particular prior distributions for each model parameter in the clinical model and sample from the posterior distribution using Markov Chain Monte Carlo (MCMC). There are several priors known to achieve sparsity and facilitate Bayesian variable selection, which we will discuss briefly. One option is to simply put vague Normal(0,*∞*) priors on each regression coefficient. This is equivalent to doing least squares regression and is impossible in cases where there are more variables than data points, because singular solutions arise. A natural extension is to place proper mean-zero Normal priors on the coefficients, which is equivalent to ridge regression. Although accommodating more predictors than data points and facilitating shrinkage, the type of shrinkage is linear which is not desirable in the current settings. This linear shrinkage leads to more shrinkage and thus greater bias for larger coefficients, while in this setting, we desire the opposite: less shrinkage for large (significant) coefficients and greater shrinkage for smaller (non-significant) ones. This type of non-linear shrinkage can be accomplished by various priors. One is the ‘spike and slab’ prior consisting of a mixture of a point mass at zero (the spike) and a Normal (the slab). Although this can accommodate a large number of predictors and avoids linear shrinkage, the shrinkage asymptotes to a constant which still results in attenuation of the truly large effects, something we want to avoid. In addition, computational complications and difficulties accompany the use of spike and slab priors. As we show below, all but one of our complete conditional distributions are in closed form, so we can avoid the computational difficulties associated with the spike and slab method, as well as the attenuation of large effects, by utilizing continuous shrinkage priors.

A widely known method that places a continuous sparsity prior on the regression coefficients is the Bayesian lasso [13], which is incorporated by assigning a double exponential (i.e., Laplace) prior to ***β***. When posterior modes are used as the coefficent estimates, this process yields the same solutions as Tibshirani’s lasso [14]. The Bayesian lasso has proven to perform well in conducting adaptive shrinkage-induced sparsity, but the single hyperparameter formulation does not allow for enough flexibility to estimate the true size of potentially large, non-zero effects. Instead, these effect estimates are shrunk toward zero along with the smaller effects [11]. An alternate class of priors we use and discuss is the Normal-Gamma (NG) prior distribution for ***β***. Incorporating this continuous prior not only provides shrinkage of the coefficients but the extra hyperparameter in the NG prior construction facilitates more adaptability in the estimated shrinkage relative to the Bayesian lasso [13] - with the NG, the larger effects are shrunk less than the smaller effects [15], thus leading to improved estimation [11]. In summary, the NG prior is extremely advantageous in our situation, since it delivers the sparsity we need, while leaving larger effects mostly unshrunk, thus aiding our estimation of the important effects.

For our method, we assign a Normal-Gamma (NG) prior distribution for each ***β***_*j*_. Our complete hierarchical clinical model can be written as:

$$\begin{array}{lll} \;Y & =\text{Normal}(X\beta ,\sigma^{2}I_{n});\; & \;\\ \;\beta & =\text{Normal}(0_{\tilde{p}},D_{\psi })\;\text{where}\;\; & \;\\ \;D_{\psi } & =\text{diag}(\psi_{1,1},\ldots ,\psi_{1,p_{1}},\ldots ,\psi_{J,1},\ldots \psi_{J,p_{J}});\; & \;\\ \;\psi_{j,i} & =\text{Gamma}(\lambda_{j},1/(2\gamma_{j}^{2}))\; & \;\\ \;\sigma^{2} & =\text{InverseGamma}(a,b),\; & \;\\ \;\lambda_{j} & =\text{Exponential}(c),\; & \;\\ \;\gamma_{j}^{-2} & =\text{Gamma}(ã,\tilde{b}/(2\lambda_{j})),\; & \; \end{array}$$

where $\tilde{p}=\sum_{j=1}^{J}p_{j}$ is the total number of predictors in the model. (Note that the double exponential prior of the Bayesian lasso would be constructed by assigning *β*_*j*,*i*_|*ψ*_*j*,*i*_ ∼ Normal(0,*ψ*_*j*,*i*_) and *ψ*_*j*,*i*_ ∼ Exponential(*λ*_*j*_). The single parameter in the exponential prior (*λ*_*j*_) is the reason such a construction has limited flexibility as compared to the NG prior which is parameterized by both *λ*_*j*_ and *γ*_*j*_.) With the NG formulation as given above, the complete conditionals for most parameters are available in closed form - we can use Gibbs sampling to update all parameters except *λ*_*j*_, which we update using a Metropolis-Hastings random walk step. More details for drawing MCMC samples are available in Appendix B.

### 2.3 Gene selection

Given the posterior samples from the MCMC, we determine which genes are significantly related to clinical outcome using a method based on the median probability model [16]. First, we define a minimum effect size which is driven by practical considerations. Since we are analyzing survival data, we use AFT models using log(survival) as the response; thus, a *δ*-fold or larger change in survival for a unit increase in a predictor corresponds to a *β*_*j*,*i*_ outside the region (log(1 - *δ*), log(1 + *δ*)), where *β*_*j*,*i*_ is the regression coefficient for platform *j* of gene *i*. Denote this region $\left(\delta_{-}^{*},\delta_{+}^{*}\right)$. (In our following analyses, we use *δ* = 0.05 which corresponds to a 5% change in survival time.) If *S* is the number of MCMC samples and $\beta_{j,i}^{\left(s\right)}$ is the *β*_*j*,*i*_ sample from iteration *s*, then $p_{+}(x_{j,i})=\sum_{s=1}^{S}I(\beta_{j,i}^{\left(s\right)}>\delta_{+}^{*})/S$ is the posterior probability that *β*_*j*,*i*_ is higher than the practical cutoff $\delta_{+}^{*}$. Similarly, $p_{-}(x_{j,i})=\sum_{s=1}^{S}I(\beta_{j,i}^{\left(s\right)}<\delta_{-}^{*})/S$ is the posterior probability that *β*_*j*,*i*_ is lower than the practical cutoff $\delta_{-}^{*}$. We flag a gene as ‘significant’ if *p*_+_(*x*_*j*,*i*_) > 0.5 or if *p*_−_(*x*_*j*,*i*_) > 0.5.

Algorithm 1 provides a concise summary of implementing the multivariate iBAG model and conducting gene selection.

Algorithm 1 Method implementation

## 3 Simulation

We investigate the shrinkage properties of our Bayesian penalized regression formulation of the clinical model as compared to least squares regression, Bayesan lasso, frequentist lasso, and frequentist elastic net through a simulation. We simulate a training dataset with 90 predictors (*J* = 3 platforms with *p*_1_ = *p*_2_ = *p*_3_ = 30 predictors from each), where 30 randomly selected *β*_*j*,*i*_’s are set exactly to 0 and the other 60 are sampled from a Laplace(*μ* = 0, *b* = 1/7) distribution; this reflects the effective sparsity we expect to see in our data. The other settings for the simulated data are *n* = 100, *σ*^2^ = 1, each *X* entry is from Normal(0,1), and **Y** = Normal(*X****β***,*σ*^2^**I**_*n*_). The test dataset used to assess performance is simulated with the same settings as the training data, but *n* = 400. We applied our method for estimating the parameters in the clinical model, using 10,000 iterations of the Gibbs sampler with 500 for a burn-in period. For both the frequentist lasso and elastic net, we ran the simulation with two standard choices for the penalty parameter *λ*: (1) ‘1 SE’ where we used the largest *λ* with cross validation error within one standard error of the minimum cross validation error and (2) ‘min’ where we used the *λ* with minimum error (from cross validation). For elastic net, we set the mixing parameter (that controls the mixture of penalties) to 0.5. The results of our method are compared to those of the other methods in Table 1.

**Table 1:** Simulation results

	$\hat{\sigma^{2}}$	**95% CI**	**90% CI**	**MSE ratio**	**MSE ratio**
		**coverage**	**coverage**	**(train data)**	**(test data)**
Our method	0.9073	0.9778	0.8889	0.2827	9.4630
Maximum likelihood	0.1181	1.00	0.9667	1	1
Bayesian lasso	0.6407	0.9667	0.9111	0.3727	8.858
Freq. lasso (1 SE)	1.2020	NA	NA	0.0983	8.1163
Freq. lasso (min)	0.6379	NA	NA	0.1851	8.8374
Freq. EN (1 SE)	0.9278	NA	NA	0.1273	8.4439
Freq. EN (min)	0.7012	NA	NA	0.1684	8.7154

Freq. EN means freqentist elastic net, which was run with mixing parameter (for penalty mixture) 0.5. The estimate of ***σ***^***2***^ is the posterior mean for our method and the Bayesian lasso. For the others, it is the mean sum of squared error. ‘CI’ is credible interval for Bayesian methods and confidence interval for frequentist methods. Note that for the frequentist lasso and elastic net, it is not possible to obtain standard errors for the coefficients set to 0, and therefore, we cannot construct the CI’s. The penalty choice of ‘1 SE’ means we used the largest parameter with error within one standard error of the minimum error, while ‘min’ means we used the parameter with minimum error (from cross validation). MSE ratio is the mean squared error from least squares divided by the MSE from the respective method. NA indicates not applicable.

We see that our method gives a good estimate of *σ*^2^ (recall *σ*^2^ = 1). We also note that the least squares regression yields coverage probabilities that are too high, while the frequentist coverage probabilities of the Bayesian credible intervals are close to the nominal levels. (Note that for the frequentist lasso and elastic net, it is not possible to obtain standard errors for the coefficients set to 0, and therefore, we cannot construct the CI’s.) For all methods (other than least squares), the MSE ratio is less than 1 for the training data but much greater than 1 for the test data; this is consistent with the idea that in this high dimensional setting with expected sparsity, least squares tends to overfit the training data, while methods that perform shrinkage lead to improved estimation on the test data and thus yield results more applicable to the overall population. Considering that the MSE ratio is the mean squared error from least squares divided by the MSE from the respective method, we see that our method has the best (largest) MSE ratio on test data, which for our purposes is the most relevant comparison criterion.

**Figure 2:**
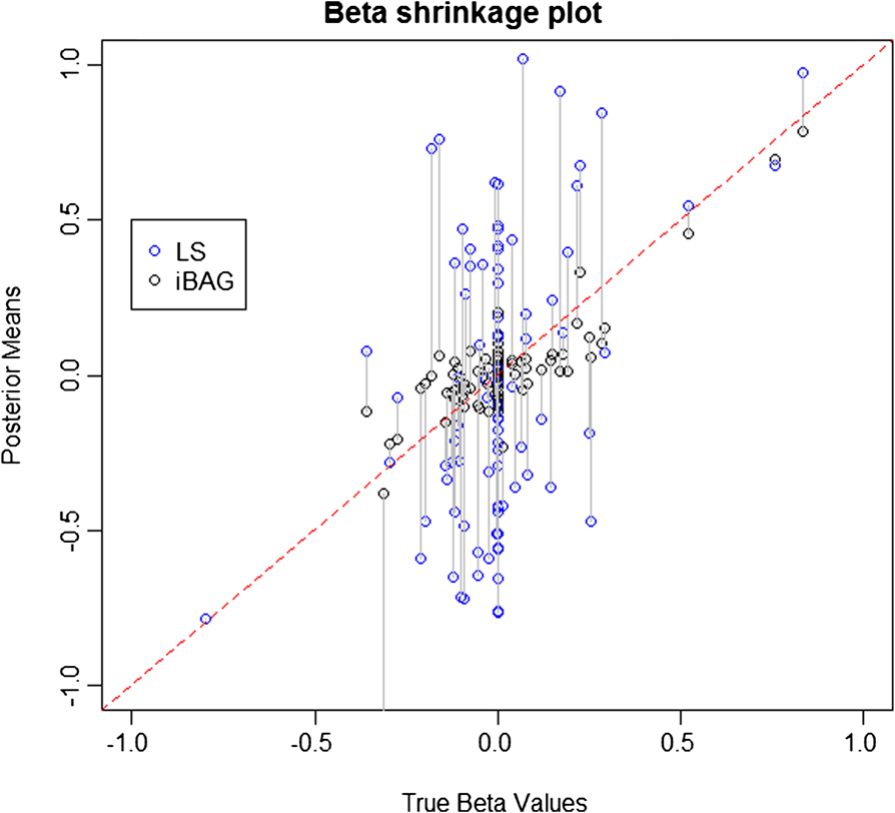
**Simulation results.** Least squares estimates and posterior means from our method are plotted against the true *β* values. The vertical lines denote the difference between the estimates from each method thus indicating the shrinkage properties of the NG prior.

We also see excellent shrinkage properties of our method in Figure 2; most least squares coefficient estimates (which are the maximum likelihood estimates) are far from the true parameter values, while the posterior means from our method shrink these estimates closer to the true values. The non-linear shrinkage and flexibility provided by the NG prior facilitate more shrinkage near 0 without severe attenuation of the estimates for truly large regression coefficients.

## 4 Integrative analysis of GBM data

GBM is one of the most common and most malignant brain tumors. The American Cancer Society estimates that in the year 2013, there will be 23,130 new cases of brain and other nervous system cancers in the USA and that 14,080 Americans will die from such cancers [17]. GBM tumors make up 17% of all primary brain tumors [18], and prognosis is typically very poor; a study with 7,259 patients, each diagnosed with GBM from 2005 to 2008, found a median survival time of 14.6 months for patients who received tumor-directed surgery and radiation therapy and a median survival time of 2.9 months for patients who did not receive any radiation treatment [19]. Treatment options include surgery, radiation, and/or chemotherapy, but even for a patient receiving more than one of these treatments, the outlook is dismal at best. Finding prognostic biomarkers related to cancer development and patient survival is an important issue, and GBM was one of first cancers to be studied in TCGA. The data currently available contains information from multiple molecular platforms (genomic/epigenomic/transcriptomic) as well as clinical data on several hundred tumor samples (approximately 500).

The availability of such extensive genomic data has prompted several studies using the TCGA GBM data, and fortunately, there continue to be discoveries of biomarkers that aid in predicting survival and identifying subtypes of GBM. One such study conducted by Verhaak et al. combined gene expression data from multiple types of microarray assays to classify tumors into four distinct subtypes (each responding differently to therapy) and to discover which gene expression levels had a significant impact on the classification. Other platforms were also used, such as copy number and mutations, in separate analyses to test for associations with subtype [20]. Another study by Noushmehr et al. used the available GBM DNA methylation data to identify a subgroup of GBM tumors associated with a significantly longer survival time [21]. In our integrative analysis, we use 163 matched tumor samples that have been assayed by expression, methylation, and copy number platforms as described below. Each of these samples has an uncensored survival time (in days), and our aim is to identify prognostic biomarkers.

### 4.1 Description of data

Our copy number data is level 2 data from the HG_CGH_244A platform; it is the normalized signal for copy number alterations of aggregated regions per probe. Our methylation data is level 3 data from the HumanMethylation27K arrays; it is the methylated sites along a gene (probe level data). Our expression data is level 3 data (summarized per gene) from the Affymetrix profiled HT_HG_U133A platform [22].

We focus our analysis on data corresponding to 49 genes implicated in important signaling pathways in GBM (RTK/PI3K, P53, and RB pathways [2]), using the following structure: 

1. *OurSurvival* (163 × 1), containing days of survival after diagnosis for each patient.

2. *OurMRNA* (163 × 49), containing mRNA expression levels for each gene (columns) for each patient (rows).

3. *OurMeth* (163 × 176), containing data on the methylation markers (columns) for each patient (rows). There can be multiple (ranging from 1 to 21) methylation markers per gene, and the columns are ordered by gene.

4. *OurCopyNumber* (163×524), containing copy number data (columns) for each patient (rows). Again, there are multiple (ranging from 1 to 43) values per gene, and the columns are ordered by gene.

One gene has no methylation data, so we remove that column from the *X* matrix, which essentially sets M_*i*_ to be 0 for that gene. Any effect that may be due to methylation for that gene would then be captured by the ‘other’ predictor in the clinical model. After standardizing the predictors and imputing the (few) missing values, we model the data using an AFT model with log survival times as the outcome and apply our method of estimating the parameters of the iBAG model.

### 4.2 Results using iBAG model

**Figure 3:**
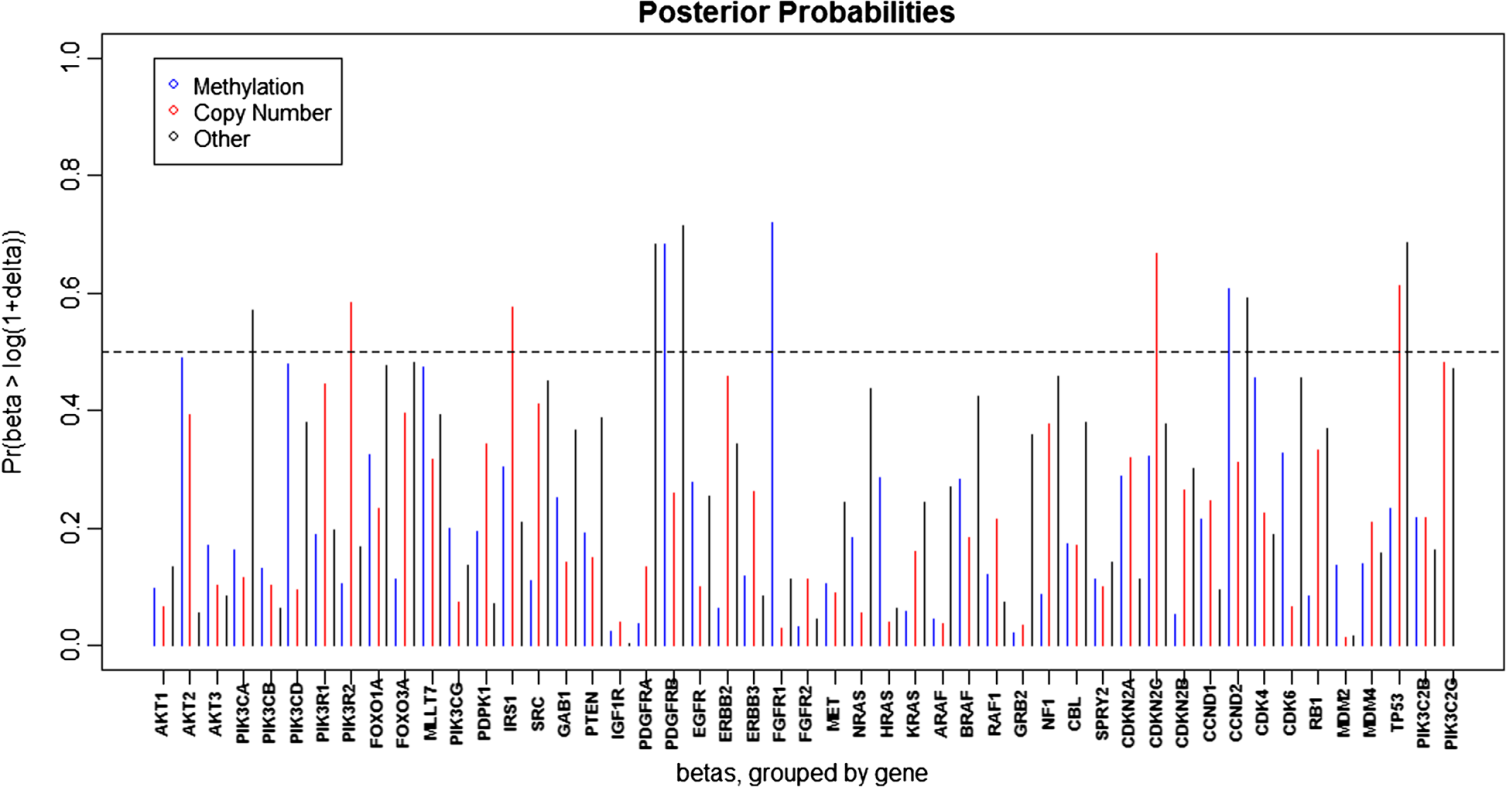
**GBM data results.** The posterior probabilities (based on MCMC samples) that *β*_*j*,*i*_ > *δ* + ∗ is plotted, where *β*_*j*,*i*_ is the clinical model regression coefficient for the marker associated with platform *j* of gene *i*, and $\delta_{+}^{*}=\log(1+\delta )$ is the transformed upper practical cutoff. For our analysis, we use *δ* = 0.05, which corresponds to a 5% change in survival time, so the posterior probability shown here indicates the probability that a one unit increase in the marker results in at least a 5% increase in survival time. We consider the marker *j*,*i* to be significant if this probability is greater than 0.5.

**Figure 4:**
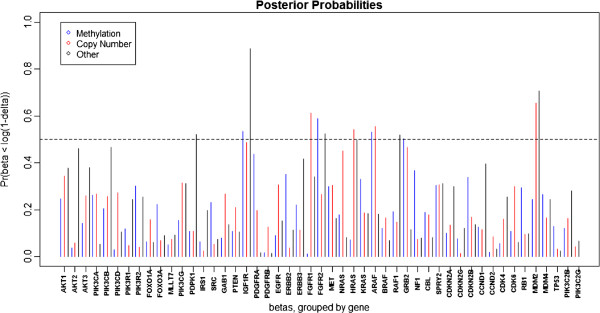
**GBM data results.** The posterior probabilities (based on MCMC samples) that $\beta_{j,i}<\delta_{-}^{*}$ is plotted, where *β*_*j*,*i*_ is the clinical model regression coefficient for the marker associated with platform *j* of gene *i*, and $\delta_{-}^{*}=\log(1-\delta )$ is the transformed lower practical cutoff. For our analysis, we use *δ* = 0.05, which corresponds to a 5% change in survival time, so the posterior probability shown here indicates the probability that a one unit increase in the marker results in at least a 5% decrease in survival time. We consider the marker *j*,*i* to be significant if this probability is greater than 0.5.

**Figure 5:**
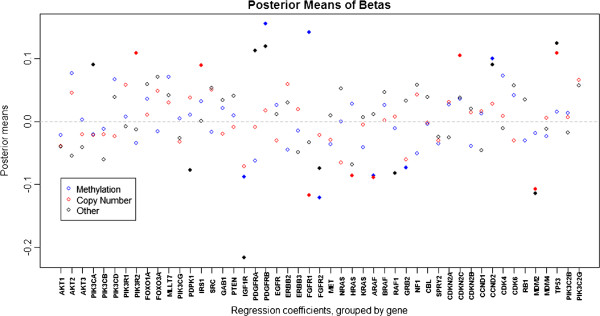
**Regression coefficient posterior means.** The estimates of the regression coefficients in the clinical model (*β*_*j*,*i*_’s) are shown, where *β*_*j*,*i*_ is the coefficient for the marker associated with platform *j* of gene *i*; the estimates are computed as the posterior means from our MCMC samples. The multiple platforms for each gene are labeled by color, and solid plot markers indicate that the effect was found to be significant, meaning that the posterior probability that a one unit increase in the marker results in at least a 5% change in survival time is at least 0.5.

After applying our method to the GBM data, we then use the method discussed in Section 2.3 to determine the significant markers using *δ* = 0.05 (corresponding to a 5% change in survival time). Figures 3 and 4 show the posterior probabilities of the effect (*β*_*j*,*i*_) being greater than $\delta_{+}^{*}$ and less than $\delta_{-}^{*}$, respectively. Figure 5 depicts the posterior means of the *β*_*j*,*i*_’s and also indicates which were flagged as significant. We find 25 markers to be significant, 12 with positive effects on survival (more expression attributed to that platform, better prognosis) and 13 with negative effects (more expression attributed to that platform, poorer prognosis). The genes with the 12 positive markers were PDGFRB, FGFR1, CCND2, PIK3R2, IRS1, CDKN2C, TP53, PIK3CA, and PDGFRA. The genes PDGFRB, FGFR1, and CCND2 were determined to be related to clinical outcome through methylation effects, while expressions of PIK3R2, IRS1, CDKN2C, and TP53 were related to clinical outcome through copy number. For PIK3CA, PDGFRA, PDGFRB, CCND2, and TP53, gene expression was related to clinical outcome through some other unspecified mechanism. The genes with the 13 negative markers were IGF1R, FGFR2, ARAF, GRB2, FGFR1, HRAS, MDM2, PDPK1, and RAF1. The first four were related to clinical response through methylation, while FGFR1, HRAS, ARAF, and MDM2 were related through copy number, and PDPK1, IGF1R, FGFR2, RAF1, and MDM2 were related through some mechanism other than methylation or copy number. Note that eight genes (IGF1R, PDGFRB, FGFR1, FGFR2, ARAF, CCND2, MDM2, and TP53) are found to be significant on two or more different platforms. We have not only identified 17 genes as having a significant effect on survival (Table 2), but we have also determined which platform(s) of those genes is (are) modulating the effect.

**Table 2:** Gene results

**Gene names**				
AKT1	MLLT7	EGFR	BRAF	*CCND2*
AKT2	PIK3CG	ERBB2	*RAF1*	CDK4
AKT3	*PDPK1*	ERBB3	*GRB2*	CDK6
*PIK3CA*	*IRS1*	*FGFR1*	NF1	RB1
PIK3CB	SRC	*FGFR2*	CBL	*MDM2*
PIK3CD	GAB1	MET	SPRY2	MDM4
PIK3R1	PTEN	NRAS	CDKN2A	*TP53*
*PIK3R2*	*IGF1R*	*HRAS*	*CDKN2C*	PIK3C2B
FOXO1A	*PDGFRA*	KRAS	CDKN2B	PIK3C2G
FOXO3A	*PDGFRB*	*ARAF*	CCND1	

All 49 genes appearing in the data are listed. Italic genes were identified by our method to have at least one significant marker.

### 4.3 Biological interpretation

There are a total of 17 genes found to affect the expression of glioblastoma tumors significantly. Of these, nine genes are negatively affecting the survival and nine genes are affecting the survival positively. The positive and negative prognostic markers are reviewed within the context of glioblastoma biology in this section.

*Negative prognostic markers*: Fibroblast growth factor pathway signaling is associated with significant tumor enhancement in glioblastoma [23]. Fibroblast growth factor receptors FGFR1 and FGFR2 play an oncogenic role in various tumor types and can be targeted by multiple small molecules in cancer therapy [24]. FGFR1 expression can be regulated by methylation level of the upstream CpG island [25]. Hyper-methylation of FGFR1 would provide positive effects by reducing the expression level of FGFR1 and thus appear to be affecting the survival in both ways. Insulin-like growth factor receptor 1 (IGF1R) is a well-known target to treat GBM and has been found to be associated with astrocytoma and meningioma as well [26]. It is also associated with anti-EGFR resistance in GBM and is a pan-cancer biomarker connected with many different tumor types [27,28]. MDM2 is a well-known oncogene and inhibitor of the tumor suppressor TP53. Previous studies in glioblastoma using expression and copy number platforms indicated the abnormal over-expression and amplification of MDM2 [29,30]. ARAF is a serine/threonine protein kinase of RAF family, known to stabilize the hetero-dimerization of RAF proteins, BRAF and CRAF [31]. Its role and over-expression are observed in other tumors but are not explored in the context of glioblastoma [32]. Growth factor receptor-bound protein 2 (GRB2) is involved in RAS signaling pathway and known to be associated with EGFR [33]. GRB2 is an interacting partner of EGFRvIII, a common mutated variant of EGFR in the molecular signaling of EGFR-driven glioblastoma [34,35].

*Positive prognostic markers*: The tumor suppressor gene TP53 is a positive prognostic marker as expected. The Cyclin-dependent kinase inhibitor CDKN2C, a known tumor suppressor of glioblastoma, is also identified as a positive marker [36]. Platelet-derived growth factors (PDGF) receptors PDGFRA and PDGFRB show positive survival effects, whose oncogenic role is well established in the context of glioma [37,38]. These PDGF receptors are the representative genes of the pro-neural subtype of glioblastoma [20,39]. Interestingly, the pro-neural subtype of glioblastoma is enriched in oligodendroglioma and has higher survival rates compared to other subtypes of glioblastoma [40]. The insulin receptor substrate gene IRS1 is shown to be one of the representative candidates for mesenchymal subtype of GBM with poor survival [41]. The role of IRS1 is not clear, given that we found it to be a positive marker in our analysis. Overall, the positive markers are generally enriched in the pro-neural subtype of glioblastoma, which was found to have prolonged survival [20].

## 5 Conclusions

In this article, we present a hierarchical Bayesian model that integrates data from multiple genomic platforms, incorporating information about the platforms’ biological relationships in order to better identify genes that are critical to patient survival and to additionally provide mechanistic information on the manner of their effect. In summary, the key advantages of our method include (1) multiple platforms are integrated in a single model; (2) the biological relationships between platforms are taken into account by the model; (3) high dimensional data can be handled easily, with shrinkage priors; (4) the NG prior on the predictors allows for flexible shrinkage of the parameter estimates; (5) the model can be extended to incorporate more platforms, as long as the underlying biological relationships are well understood; and (6) we have the ability to not only identify genes significant to patient survival but also gain mechanistic information on the manner by which the gene expression is related to outcome.

Applying our methodology to a GBM dataset from TCGA, our method identified several genes with effects that have a significant impact on survival time. In addition, we identified whether each gene was related to clinical outcome through methylation, copy number, or some other mechanism. This is especially advantageous in investigating the biological mechanisms of cancer development and progression, and in subsequent development of novel therapeutic strategies.

Although beyond the scope of this paper, two areas of future investigation might include (1) relaxing the parametric assumptions by using generalized additive models instead of linear models or substituting specified parametric non-linear models if they are justified by the science, and (2) dynamic modeling, which would require different types of data and further modeling assumptions to capture complex patterns of feedback loops both within and between platforms.

## Appendices

### Appendix A Data imputation

Since the percentage of missing data is so low (∼5*%* for methylation and ∼0.1*%* for copy number), we choose to do imputation using the following algorithm for both the methylation data and the copy number data: (1) For each marker, replace any NA’s with the mean of the other patients. Call this resulting matrix Temp. (2) Use Temp to calculate a correlation matrix between markers. (3) For each marker with missing value(s), regress it on the three markers which it is most highly positively correlated with (using the Temp matrix for the predictors to avoid further complications from missing data). (4) Substitute this predicted value for the missing value in the original matrix.

### Appendix B Complete conditionals

$$\begin{array}{ll} \;\beta |\text{rest}∼ & \text{Normal}\{{\left(X^{T}X+\sigma^{2}D_{\tau }^{-1}\right)}^{-1}X^{T}\text{Y},\sigma^{2}(X^{T}X\\ & \;+\sigma^{2}{D_{\tau }^{-1})}^{-1}\}\\ \;\sigma^{2}|\text{rest}∼ & \text{Inv.Gamma}(a=a+n/2,b=b+\{(\text{Y}\\ & \;-\;X{\beta )}^{T}(\text{Y}-X\beta )\}/2)\\ \;\psi_{j,i}|\text{rest}∼ & \text{Gen.Inv.Gaussian}(a=\gamma_{j}^{\;-2},b=\beta_{j,i}^{2},p=\lambda_{j}-1/2),\\ & \text{where}\;V=\text{Gen.Inv.Gaussian}(a,b,p)\\ & \text{has density}{\left(a/b\right)}^{p/2}v^{p-1}\exp\{-(\text{av}+b/v)/2\}/\\ & \{2K_{p}(\sqrt{\text{ab}})\},\text{where}\;K_{p}(\cdot )\;\text{is a modified}\\ & \text{Bessel function of the second kind.}\\ \;\lambda_{j}|\text{rest}∼\; & {\left(1/\lambda_{j}\right)}^{ã}\exp\left\{-\tilde{b}\gamma_{j}^{-2}/(2\lambda_{j})-c\lambda_{j}\right\}\\ & \times \left(\overset{p_{j}}{\underset{i=1}{\prod }}\psi_{j,i}^{\lambda_{j}}\right)/\left[{\left\{\Gamma (\lambda_{j})\right\}}^{p_{j}}{\left(2\gamma_{j}^{2}\right)}^{p_{j}\lambda_{j}}\right]\\ \;\gamma_{j}^{-2}|\text{rest}∼\; & \text{Gamma}(a=p_{j}\lambda_{j}+ã,b=(\tilde{b}/\lambda_{j}\;+\;\overset{p_{j}}{\underset{i=1}{\sum }}\psi_{j,i})/2) \end{array}$$

In the Metropolis-Hastings update step, the proposed value is $\lambda_{j}^{*}=\exp(\sigma_{\lambda }^{2}z)\lambda_{j}$ where *z* ∼ Normal(0,1) and the tuning parameter $\sigma_{\lambda }^{2}$ is chosen to result in an acceptance rate between 20% and 30%. The acceptance probability is then $\min\left\{1,\frac{\pi (\lambda_{j}^{*})}{\pi (\lambda_{j})}{\left(\frac{\Gamma (\lambda_{j})}{\Gamma (\lambda_{j}^{*})}\right)}^{p_{j}}{\left({\left(2\gamma_{j}^{2}\right)}^{-p_{j}}\prod_{i=1}^{p_{j}}\psi_{j,i}\right)}^{\lambda_{j}^{*}-\lambda_{j}}\left(\frac{\lambda_{j}^{*}}{\lambda_{j}}\right)\right\}$ where $\pi (\lambda_{j})={\left(1/\lambda_{j}\right)}^{ã}\exp\{-\tilde{b}\gamma_{j}^{-2}/(2\lambda_{j})-c\lambda_{j}\}$, the prior for *λ*_*j*_.

### Initial values and hyperparameters

The initial values and hyperparameters are chosen as follows: 

• The hyperparameters for *σ*^2^ are *a* = *b* = 0.001, so as to be uninformative.

• The hyperparameter for *λ*_*j*_ is *c* = 1 [11].

• The hyperparamters for $\gamma_{j}^{-2}$ are $\tilde{a}=2$ and $\tilde{b}$ = the mean of the least squares $\hat{\beta_{j,i}^{2}}$[11].

• The initial ***β*** is the estimate from the frequentist lasso with a single shrinkage parameter.

• The initial *σ*^2^ is the mean sum of squares from the frequentist lasso.

• Each initial *λ*_*j*_, *ψ*_*j*,*i*_, and $\gamma_{j}^{-2}$ is set to 1.

## Competing interests

The authors declared that they have no competing interests.
